# Applications of (+) Usnic Acid Modulate Antioxidant Enzymatic Activity in Strawberry Plants

**DOI:** 10.3390/molecules31132362

**Published:** 2026-07-05

**Authors:** Laura Castro-Rosalez, Antonio Juárez-Maldonado, Adalberto Benavides-Mendoza, Susana González-Morales, Elizabeth García-León, Fabián Pérez-Labrada

**Affiliations:** 1 Protected Agriculture Postgraduate Program, Universidad Autónoma Agraria Antonio Narro, Saltillo 2315, Mexico; lauracrosalez@gmail.com; 2 Department of Botany, Universidad Autónoma Agraria Antonio Narro, Saltillo 2315, Mexico; antonio.juarez@uaaan.edu.mx; 3 Laboratorio Nacional Conahcyt de Ecofisiología Vegetal y Seguridad Alimentaria (LANCEVSA), Universidad Autónoma Agraria Antonio Narro, Saltillo 2315, Mexico; abenmen@gmail.com; 4 Department of Horticulture, Universidad Autónoma Agraria Antonio Narro, Saltillo 2315, Mexico; 5 Secretaría de Ciencia, Humanidades, Tecnología e Innovación (SECIHTI), Universidad Autónoma Agraria Antonio Narro, Saltillo 2315, Mexico; qfb_sgm@hotmail.com; 6 Campo Experimental Valle del Fuerte, Instituto Nacional de Investigaciones Forestales, Agrícolas y Pecuarias, Juan José Ríos 81110, Mexico; garcia.elizabeth@inifap.gob.mx

**Keywords:** carbonic anhydrase, RuBisCO, PEP-carboxylase, catalase

## Abstract

Usnic acid (UA) is a secondary metabolite produced by lichens that has attracted interest because of its antimicrobial, photoprotective, and antioxidant properties, suggesting its potential use as a biostimulant in agriculture. However, its evaluation in agricultural crops is limited. In the present study, we evaluated the effect of applying (+) UA on enzymatic and non-enzymatic antioxidant systems, photosynthetic pigments, photosynthetic enzyme activity, and markers of oxidative stress in “Albion” strawberry plants. The plants were grown in a peat moss:perlite substrate (1:1, *v*/*v*) and cultivated under tunnel greenhouse conditions using a nutrient solution applied via fertigation. (+) UA was applied at 400 µg/mL via three routes (foliar, drench, and a combination of foliar and drench) on three occasions. Leaf tissue was collected 117 days after transplantation, and the biochemical parameters were quantified. (+) UA increased the activity of glutathione peroxidase (GPX) (53% via foliar-drench) and catalase (CAT) by 73.5% (via drench), and reduced glutathione (GSH) content by 58% (via foliar). β-carbonic anhydrase (βCA) activity increased by 415% and 384% (foliar and foliar-drench, respectively). Likewise, Ribulose 1,5-bisphosphate carboxylase-oxygenase (RuBisCO) activity increased by 58.23% (drench) and phosphoenolpyruvate carboxylase (PEPC) by 25% and 46% (foliar and drench), suggesting positive effects on the processes associated with CO_2_ assimilation and transport. In contrast, no significant changes were observed in the levels of hydrogen peroxide (H_2_O_2_), malondialdehyde (MDA), or proline, indicating the absence of oxidative stress. These findings suggest that (+) UA modulates the enzymatic antioxidant system, promoting favorable physilogical responses without inducing oxidative stress. The use of (+) UA has been proposed as a potential promoter of metabolism in agricultural crops. In addition, new avenues of research are being explored to investigate the role in modulating antioxidant responses under biotic and abiotic stress conditions.

## 1. Introduction

Secondary metabolites (SMs) are organic compounds derived from the primary metabolism of plants, fungi, bacteria, algae, and animals. Unlike primary metabolites, SMs do not directly contribute to the normal growth and development of an organism; however, they play a key role in defense and adaptation to the environment [1,2]. Some SMs have gained prominence in agriculture because of their biological properties and their use as biostimulants, signaling molecules, and resistance inducers under stressful conditions [2]. Among these compounds is usnic acid (UA), a secondary metabolite produced primarily by lichens of the genera *Cladonia*, *Alectoria*, *Usnea*, *Lecanora*, *Ramalina*, and *Evernia* [3,4]. This molecule is characterized by its low solubility in water and high solubility in organic solvents, such as ether, acetone, benzene, dimethyl sulfoxide (DMSO), and chloroform [4,5]. There are two enantiomeric forms of UA: levorotatory (−) and dextrorotatory (+), with the latter being the most studied because of its commercial availability [6].

UA possesses antimicrobial, antioxidant, anti-inflammatory, antitumor, analgesic, and photoprotective properties against UV radiation [3,4]. However, most studies have focused on clinical settings, and research on its application in agriculture remains limited. In plants, UA application induces changes in transpiration, photosynthesis, respiration, enzymatic activity, and the production of phenolic compounds [7,8,9,10]. In this regard, Lascève and Gaugain [11] reported a 60% reduction in the transpiration rate and a 40% reduction in water loss in sunflower and corn seedlings following the application of UA at a concentration of 5 × 10^−5^ M via the root system through the nutrient solution for 24 h. This effect was attributed to a decrease in stomatal conductance resulting from the partial stomatal closure. Furthermore, Prokopiev and Filippova [9] reported that application of the (+) and (−) enantiomers of UA at concentrations of 62.5–1000 µM (applied directly to filter paper during sowing) increased the activity of the enzymes superoxide dismutase (SOD), CAT, and peroxidase (POX), as well as an increase in MDA content in *Allium fistulosum* seedlings under in vitro conditions. Similarly, Kikowska et al. [10] reported an increase in the concentration of flavonoids (isoquercetin) and phenolic acids (rosmarinic acid and caffeic acid) in *Eryngium alpinum* seedlings with UA application at 3.125 µM under in vitro conditions. These results suggest that UA can promote the accumulation of antioxidant compounds in plants of agricultural interest.

However, the negative effects of UA on the physiology of some plants have been reported. For example, Latkowska et al. [12] indicated that applications of (+) UA at 30 μM in tomato plants grown in a hydroponic system caused a 41% reduction in photosynthesis and an 80% reduction in respiration rate, in addition to decreasing the content of chlorophylls (30%), carotenoids (35%), and chlorophyll a/b ratio (25%). The authors suggested that UA may interfere with the activity of the photosynthetic apparatus, including chloroplasts, and the enzymatic activity of protoporphyrin oxidase and 4-hydroxyphenylpyruvate dioxygenase, thereby disrupting the synthesis of photosynthetic pigments, which in turn reduces the light-harvesting capacity and photosynthetic efficiency. Similarly, they noted that a reduction in respiration may be associated with the disruption of electron flow during photosynthesis. However, they also noted that the negative effects of UA depend on the concentration and exposure time (30 μM, 21 days). Therefore, a hormetic response of plants to UA exposure is suggested. Positive effects were observed at low concentrations, whereas toxic effects were observed at high concentrations.

In another study, Latkowska et al. [13] reported a 58% increase in lipid peroxidation and proline content in tomato plants treated with 30 μM (+) UA in the nutrient solution for 21 d. This is because UA has been identified as an allelopathic compound. Plants exposed to stress conditions and allelopathic compounds increase the production of reactive oxygen species, which damage the fatty acids in cell membranes, causing their degradation and increasing the MDA content. In contrast, proline accumulation is an osmoprotective and antioxidant defense mechanism that helps counteract oxidative stress and is a physiological osmoprotective response. Bialczyk et al. [14] reported that the application of 30 μM UA to tomato plants under hydroponic conditions (21 days) reduced the concentration of indoleacetic acid in leaves and roots (by 61% and 66%, respectively), cytokinins (by 62% and 36%), and abscisic acid (by 40% and 71%).

It is known that UA has radiation-protective properties [15] and can potentially be synthesized in plants under drought stress conditions [16]; however, the applied concentration affects photosynthetic activity. It has recently been documented that the application of 1 mg/mL of UA inhibited the growth of *Physcomitrium patens*, as this metabolite alters the activity of ascorbate peroxidase (APX) and GPX enzymes and modifies proteins associated with photosynthesis, carbohydrate metabolism, and lipid biosynthesis [17]. Likewise, high doses of UA act as bioherbicides because they inhibit photosystem II (PSII). In this regard, applying a nanoformulation that uses ultra-small (10 nm) superparamagnetic iron oxide nanoparticles as a smart delivery system for UA in lettuce enhances PSII inhibition and increases oxidative stress [18].

Similarly, molecules with a central UA structure (acylhydrazide-modified usnic acid derivatives) exhibit antifungal activity against *Thanatephorus cucumeris* and *Alternaria solani* by suppressing the peroxidase and catalase activities of the pathogens, accumulating ROS, changing membrane potential, and altering redox homeostasis [19]. In another study, Atıcı et al. [20] documented that the application of UA to *Eruca sativa* Mill. microgreen growth (stem length, root length, and fresh and dry weights). Interestingly, the authors found that high doses (8 and 10 mg/L) acted as promoters, whereas low doses (2 mg/L) showed an inhibitory effect. Moreover, applying 12.5 μg/mL of (+) UA to *Solanum lycopersicum* does not induce toxic effects or interfere with biomass accumulation [21]. This finding suggests a knowledge gap regarding the hormetic response of UA in different plants and the route of application, necessitating further studies to provide information on this topic. In this regard, free UA decreases microbial carbon biomass, acid protease and acid phosphatase enzyme activity, as well as the abundance of the eukaryotic 18S ribosomal RNA gene [22], suggesting that the response to UA depends on the route of application.

Considering that nearly 18,000 lichen species have been recognized worldwide, there is substantial potential for the availability of raw materials for the production of UA, which typically accounts for 2–8% of the dry thallus weight. Furthermore, the increasing implementation of lichen cultivation systems [23] could significantly contribute to the sustainable production of this metabolite. The same authors noted that both acetone and methanol are typically the most common solvents used to obtain this metabolite, but new molecules and extraction methods are being studied to scale up the process and apply it in agriculture. Similarly, nanotechnology offers opportunities to improve UA efficiency [22]. The growing interest in the discovery and use of natural compounds with biostimulant properties that improve agricultural productivity suggests that UA may be a promising alternative. Therefore, this study aimed to evaluate the effects of (+) UA application via foliar spray, drench, and foliar-drench on photosynthetic, antioxidant, and oxidative stress indicators in strawberry plants grown under greenhouse conditions, based on the hypothesis that UA can induce a favorable physiological response by stimulating the antioxidant system without causing oxidative stress.

## 2. Results and Discussion

### 2.1. Photosynthetic Pigments

The results for the photosynthetic pigments are presented in Table 1. The contents of chlorophyll (Chl) a, b, total chlorophyll, β-carotene, and yellow pigments showed no significant differences among the treatments (*p* > 0.05). The Chl a/Chl b ratio decreased by 11% in plants receiving foliar application of UA (UAF) compared to the control. It has been reported that (−) UA interferes with protoporphyrin oxidase and 4-hydroxyphenylpyruvate dioxygenase, which are associated with the synthesis of chlorophylls and carotenoids [8]. Latkowska et al. [12] suggest that (+) UA under high irradiance conditions can promote chlorophyll photoinhibition by blocking electron flow in the thylakoid membranes, a process associated with the sequestration of Mn^2+^ ions and a reduction in carotenoid content.

In this study, irradiance levels were not high (average 650 ± 38 µmol m^−2^ s^−1^), and the application of (+) UA did not induce significant changes in the content of these pigments. These findings differ from those reported by Latkowska et al. [12], who reported that (+) UA reduced the content of chlorophyll (30%), carotenoids (35%), and the chlorophyll a/b ratio (25%) in tomato plants. These differences in responses may be associated with the plant species, enantiomeric form, route of application, and duration of exposure to the metabolite. Therefore, it is suggested that in strawberry plants, the application of (+) UA does not affect photosynthetic pigment content. The reduction in the Chl a/Chl b ratio may be associated with adjustments in the photosynthetic apparatus. Furthermore, it has recently been documented that plants under stress may synthesize this metabolite [16], so its exogenous application, depending on the dose administered, could have beneficial effects on the photosynthetic machinery.

### 2.2. Non-Enzymatic Antioxidants

**Figure 1:**
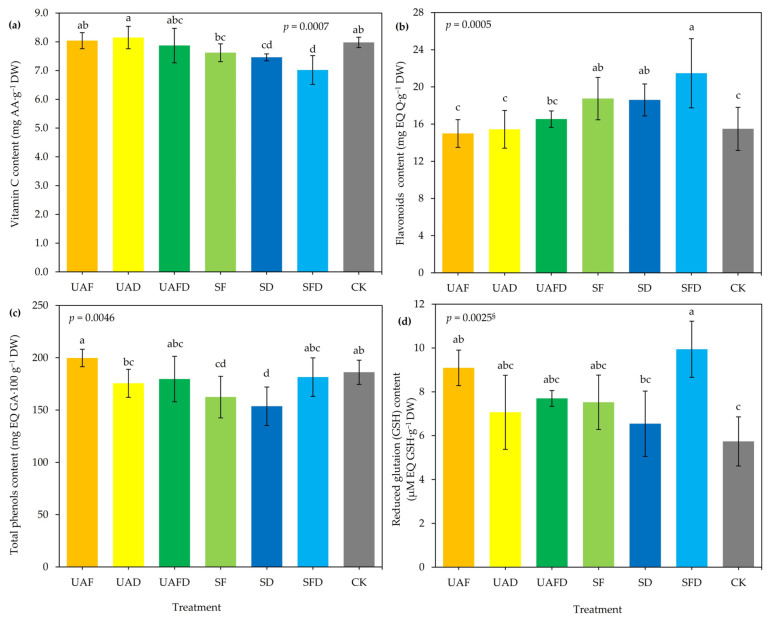
Content of non-enzymatic antioxidants in leaf tissue of the “Albion” strawberry variety treated with usnic acid. (**a**) Vitamin C content; (**b**) Flavonoid content; (**c**) Total phenol content; (**d**) Reduced glutathione (GSH) content. UAF = 400 µg/mL of usnic acid applied foliar, UAD = 400 µg/mL of usnic acid applied drench, UAFD = 400 µg/mL of usnic acid applied foliar + drench, SF = solvent (40% NaOH) applied foliar, SD = solvent (40% NaOH) applied drench, SFD = solvent (40% NaOH) applied drench + foliar, CK = control with distilled water. Data are means ± standard deviation (SD) of five replicates. Different letters in the same vertical bar indicated significant differences according to Fisher’s Least Significant Difference (LSD) test (*p* < 0.05). ^§^ Non-parametric variable (Kruskal–Wallis’s test). Different letters in the same vertical bar indicated significant differences according to Dunn’s Pairwise Comparison post hoc test (*p* < 0.05).

Vitamin C (ascorbic acid), flavonoids, phenols, and GSH are part of the non-enzymatic antioxidant system in plants. These compounds regulate free radical levels, thereby protecting cells from oxidative stress-induced damage [24]. The levels of these antioxidants showed variable responses depending on the treatment (Figure 1a–d). However, UA application did not significantly increase vitamin C levels. This suggests the need to evaluate higher doses or more frequent applications of this metabolite. In contrast, the solvent (SFD) reduced vitamin C content by 12% (*p* < 0.05) compared to that in CK. This response could be related to the susceptibility of strawberry plants to the presence of Na^+^ in the solvent, which could trigger the accumulation of reactive oxygen species (ROS) and cause oxidative stress [25]. The increase in ROS may have led to higher vitamin C intake as a protective mechanism, given its role as an electron donor that helps neutralize ROS by converting them into less-harmful compounds [26].

The flavonoid concentration in strawberry leaf tissue was significantly higher in the SFD, SF, and SD treatments (*p* < 0.05), whereas the application of UA (UAF, UAD, and UAFD) yielded results similar to those of the control (Figure 1b). This contrasts with the findings reported by Kikowska et al. [10], in which UA applications increased the flavonoid content (isoquercetin) in *Eryngium alpinum* plants. As mentioned, the increase in flavonoids in SFD, SF, and SD could be associated with oxidative stress generated by excess Na^+^, although in this case, the plants likely attempted to mitigate stress by increasing flavonoid production. Furthermore, this could be related to the decrease in vitamin C in SFD, SF, and SD as a compensatory response, in which the plants possibly used vitamin C as a substrate to increase flavonoid synthesis, thereby strengthening their antioxidant defense against excess Na^+^ [26]. Similarly, the increase in flavonoid content observed in the SFD, SF, and SD treatments may be associated with the involvement of these compounds in maintaining Na^+^/K^+^ balance in plants under the additional Na^+^ load supplied by the solvent [27].

Regarding total phenols, SF and SD treatments reduced phenolic levels by 12.75% and 17.46%, respectively, relative to CK, whereas UAF, UAFD, and SFD showed similar behavior (Figure 1c). The decrease observed with solvent application (foliar and drench) may be explained by disturbances in phenolic metabolism, including a reduction in phenylalanine ammonia-lyase activity, which results in lower phenolic biosynthesis.

In the case of GSH content (Figure 1d), a significant increase of 73.32% was observed under the SFD treatment, followed by the UAF treatment with 58.5% increase, compared to CK (*p* < 0.05). The UAFD and SF treatments increased this parameter by 34.19% and 31.15%, respectively, compared with the control treatment. Since the solvent used contained ~100 mM NaOH, its application to strawberry leaf tissue may have generated free radicals that acted as signaling molecules, promoting an increase in GSH as a detoxification pathway, an effect that was potentiated by UA. This suggests that the decrease in vitamin C and the increase in GSH are part of the plant’s antioxidant response, activating the ascorbate-glutathione pathway (Foyer-Halliwell-Asada cycle) to counteract Na^+^ toxicity [28].

Taken together, these results indicate that UA does not negatively impact the antioxidant content in strawberry plants, and that changes in the content of these compounds may be related to (i) oxidative stress induced by Na^+^ and (ii) an initial activation of the antioxidant system by UA, as documented in recent studies [18].

Therefore, alternatives to NaOH should be evaluated as UA solvents. In an effort to identify alternative solvents, several studies have shown that acetone, natural eutectic solvents (a mixture of thymol and camphor) [29], methanol, n-hexane, ethyl acetate, ethanol, chloroform, and their respective mixtures are effective and potentially safe for use in agriculture [23,30]. However, owing to its polarity [31] and lipophilic nature, usnic acid has low solubility in polar solvents [29]; therefore, it is suggested to standardize the use of the aforementioned solvents.

### 2.3. Enzymatic Antioxidants and PAL Activity

**Figure 2:**
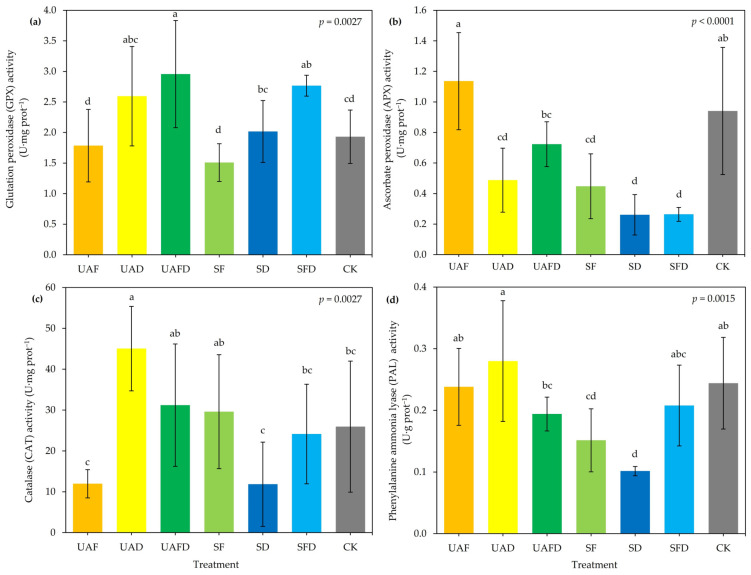
Enzymatic antioxidant content and PAL activity in leaf tissue of the “Albion” strawberry variety treated with usnic acid. (**a**) glutathione peroxidase (GPX); (**b**) ascorbate peroxidase (APX); (**c**) catalase (CAT): (**d**) phenylalanine ammonia lyase (PAL). UAF = 400 µg/mL of usnic acid applied foliar, UAD = 400 µg/mL of usnic acid applied drench, UAFD = 400 µg/mL of usnic acid applied foliar + drench, SF = solvent (40% NaOH) applied foliar, SD = solvent (40% NaOH) applied drench, SFD = solvent (40% NaOH) applied drench + foliar, CK = control with distilled water. Data are means ± standard deviation (SD) of five replicates. Different letters in the same vertical bar indicated significant differences according to Fisher’s Least Significant Difference (LSD) test (*p* < 0.05).

The enzymes GPX, APX, and CAT are part of the enzymatic antioxidant system in plants, which constitutes the primary protective mechanism for regulating ROS levels, such as H_2_O_2_ and O_2_ [32,33]. In this study, UA application significantly increased the activity of these enzymes, suggesting stimulation of the antioxidant system. UAFD treatment increased GPX activity (Figure 2a) by 53% compared to CK (*p* < 0.05), and a similar effect was observed with SFD treatment. In the case of APX (Figure 2b), the application of UAF promoted greater activity than that of the other treatments but was not significantly different from that of CK. CAT activity (Figure 2c) increased by 73.5% in the UAD treatment group (*p* < 0.05). These results are consistent with previous reports linking UA to the stimulation of GPX and CAT activities [34,35]. Latkowska et al. [13] reported a 58% increase in CAT activity in tomato roots treated with (+) UA at 30 µM. The increase in the activity of these enzymes suggests that UA acts as a modulator of the enzymatic antioxidant system and may be used to strengthen the ability of plants to respond to stress conditions.

PAL activity increased by 14.72% in plants treated with UAD. In contrast, the SF and SD treatments significantly reduced the activity of this enzyme by 37.97% and 58.44%, respectively, compared to CK. Similarly, the combined application (foliar + drench) of UA and the solvent resulted in reductions of 20.49% and 14.81%, respectively. Finally, UAF reduced PAL activity by 2.43% (Figure 2d). PAL is the enzyme responsible for activating the phenylpropanoid pathway, which leads to the synthesis of antioxidant and protective compounds such as phenolic acids and flavonoids [36]. However, although UA application stimulated PAL activity, no significant increase in the content of these metabolites was observed (Figure 1b,c). This could indicate that PAL activation did not specifically result in the accumulation of these compounds and that its activity was likely directed toward alternative pathways for the production of other protective substances or toward the redistribution of plant metabolism [36]. Another possibility is that UA acts solely as a promoter of this pathway without causing significant damage to plants, triggering the overproduction of flavonoids and phenolic compounds. These results are interesting as they suggest new areas of opportunity for evaluating the effect of this metabolite in plants exposed to different types of stress and its potential role in modulating the defensive metabolic pathways.

### 2.4. Enzyme Activity Associated with Photosynthesis

The activities of RuBisCO, PEP carboxylase, and βCA associated with photosynthesis are shown in Table 2. RuBisCO is the enzyme responsible for carbon fixation during photosynthesis and is a key factor influencing crop yield [37]. UAD treatment induced a ~58% increase in RuBisCO activity compared with CK (*p* < 0.05). In contrast, plants treated with the solvent (foliar and drench applications) showed a reduction (by 62.84% and 68.97%, respectively) in RuBisCO activity; this decrease could be related to the Na^+^ content in the solvent, as high salinity levels can alter the enzyme’s structure, causing the separation of its subunits and, consequently, the inhibition of its activity [38]. Likewise, salinity can exert indirect effects by reducing the availability and solubility of CO_2_, which negatively impacts RuBisCO performance [38]. However, the data obtained in this study show that UA application enhances this enzyme, which may suggest improved CO_2_ fixation and, potentially, biomass accumulation.

Similarly, SF and SD treatments reduced PEP carboxylase activity (~72.5%) compared to the control (*p* < 0.05), whereas UA increased the activity of this enzyme by 25.3% and 46.3% when applied via foliar spray and drench, respectively. In C3 photosynthetic plants, such as strawberries, PEP carboxylase performs anaplerotic functions by supplying carbon to the tricarboxylic acid cycle (Krebs cycle), contributing to the synthesis of organic acids and the regulation of carbon flux and nitrogen assimilation, as well as participating in the response to biotic and abiotic stress [39,40]. The increase in this enzyme following urea application could suggest a metabolic regulation in strawberry plants that adjusts the rate of cellular respiration and its anaplerotic balance. Several studies have documented an increase in PEP carboxylase activity under high-salinity conditions in response to stress [40,41,42]. This phenomenon, observed during solvent treatments, may be associated with an ionic imbalance caused by sodium accumulation.

βCA increased by 415% and 384% in plants with UAF and UAFD, respectively (*p* < 0.05). βCA participates in the conversion of CO_2_ to bicarbonate (HCO_3_^−^) and H^+^ [CO + H_2_O = H_2_CO_3_ → HCO_3_^−^ + H^+^) and is also involved in gas exchange between plants and the atmosphere and has been implicated in stress responses [43]. The increase in βAC suggests stimulatory effects on processes related to CO_2_ conversion, storage, and transport. These results suggest a selective effect of UA on the photosynthetic enzymes. Furthermore, no signs of UA toxicity were observed, as no significant alterations were noted in either enzymatic activity or photosynthetic pigment content (Table 1).

Due to its structure (a dibenzofuran composed of 18 carbon atoms, three of which belong to the carbonyl group C=O) [44], UA affects photosynthetic activity [15] in a dose-dependent manner, as high concentrations alter the electron flow at the interface between quinone A (QA) and quinone B (QB) and, by increasing oxidative stress, promote the activation of SOD and CAT enzymes [18] in addition to altering bioactive metabolites such as glycine, serine, palmitoleic acid, α-linolenic acid, linoleic acid, oleic acid, stearic acid, 1-monopalmitin, glycerol monostearate, neophytadiene, and α-tocopherol [45].

**Figure 3:**
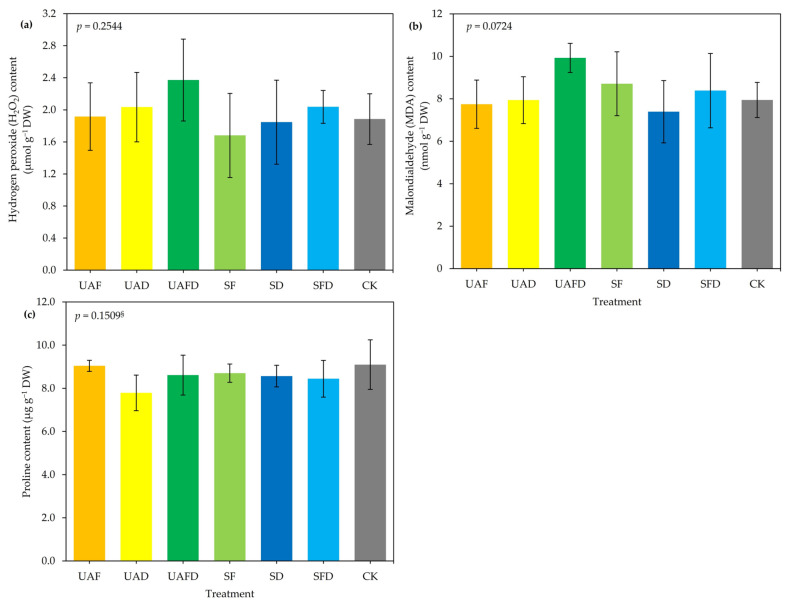
Stress indicators, including (**a**) hydrogen peroxide (H_2_O_2_); (**b**) malondialdehyde (MDA), and (**c**) proline, were measured in strawberry plants treated with usnic acid. UAF = 400 µg/mL of usnic acid applied foliar, UAD = 400 µg/mL of usnic acid applied drench, UAFD = 400 µg/mL of usnic acid applied foliar + drench, SF = solvent (40% NaOH) applied foliar, SD = solvent (40% NaOH) applied drench, SFD = solvent (40% NaOH) applied drench + foliar, CK = control with distilled water. Data are means ± standard deviation (SD) of five replicates. ^§^ Non-parametric variable (Kruskal–Wallis’s test).

In this regard, the improvement in RuBisCO and βCA activity, as well as the change in PEPC, suggests that the strawberry’s carbon fixation mechanism is enhanced without affecting its redox state, as indicated by the behavior of the enzymes (Figure 2) and stress indicators (Figure 3).

### 2.5. Oxidative Stress Indicators

The damage associated with oxidative stress is illustrated in Figure 3. The levels of H_2_O_2_, MDA, and proline did not show significant changes in the plants treated with the different treatments (*p* > 0.05). These results suggest that adjustments in the content of non-enzymatic antioxidants and antioxidant enzymatic activity were effective in mitigating the stress caused by the presence of Na^+^ in the solvent. In the case of UA, it is suggested that it did not induce oxidative stress in the plants, contrary to the findings of Latkowska et al. [13], who reported an increase in MDA and proline levels in UA-treated tomato plants. This may be related to the duration of exposure and the concentration of the metabolite used (hormetic effect). This is of vital interest, as recent studies have shown that the application of medium-to-high doses (8 and 10 mg/L) improves growth and biomass accumulation, whereas low doses (2 mg/L) inhibit these parameters in *Eruca sativa* Mill. These findings suggest that usnic acid may act as a natural biostimulant [20]. Meanwhile, applying 12.5 μg/mL of (+) UA to *Solanum lycopersicum* does not induce toxic effects or interfere with biomass accumulation [21]. In line with the specialized literature, the data obtained in this study suggest that the hormetic response of UA varies considerably depending on the crop; this information gap calls for further research on various agriculturally important plants, such as strawberries, to facilitate the potential standardization of its use in the future.

### 2.6. Principal Component Analysis (PCA)

PCA was used to examine the relationship between usnic acid treatments and biochemical, photosynthetic, enzymatic, and non-enzymatic antioxidant parameters, as well as stress indicators in strawberry plants.

**Figure 4:**
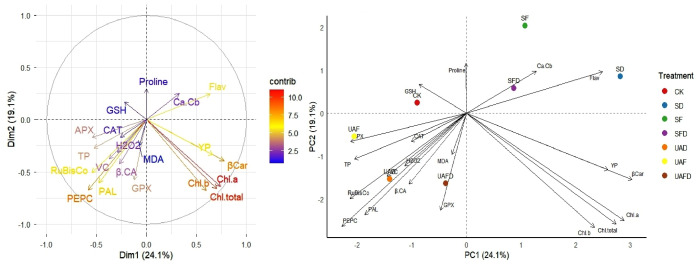
Principal component analysis of variables associated with photosynthesis, antioxidants, and oxidative stress in the leaf tissue of UA-treated “Albion” strawberry plants at 400 µg/mL. APX = ascorbate peroxidase, Ca.Cb = chlorophyll a/b ratio, CAT = catalase, Chl.a = chlorophyll a, Chl.b = chlorophyll b, Chl.total = total chlorophyll, Flav = flavonoids, GPX = glutathione peroxidase, GSH = reduced glutathione, H2O2 = hydrogen peroxide, MDA = malondialdehyde, PAL = phenylalanine ammonia lyase, PEPC = Phosphoenolpyruvate carboxylase, RuBisCO = Ribulose 1,5-bisphosphate carboxylase-oxygenase, TP = total phenol content, VC = Vitamin C, YP = yellow pigments, β.CA = β-Carbonic anhydrase, βCar = β-carotene, UAF = 400 µg/mL of usnic acid applied foliar, UAD = 400 µg/mL of usnic acid applied drench, UAFD = 400 µg/mL of usnic acid applied foliar + drench, SF = solvent (40% NaOH) applied foliar, SD = solvent (40% NaOH) applied drench, SFD = solvent (40% NaOH) applied drench + foliar, CK = control with distilled water.

The PCA (Figure 4) identified two components that together explained 43.2% of the total variability. The treatments showed clear separation from each other. Component 1 was associated with a gradient of physiological variations. The variables positively associated with this component were β-carotene, chlorophylls, flavonoids, and yellow pigments (photosynthetic activity), whereas those negatively associated with this component were PEPC, RuBisCO, APX, and PAL (enzyme systems). In other words, there is a gradient between accumulated pigments and enzymatic metabolism in strawberry plants. The second principal component represented secondary variation related to oxidative stress and antioxidant metabolism, where the variables proline, flavonoids, GSH, and the chlorophyll a/b ratio showed positive values, while chlorophylls (a, b, total), PEPC, PAL, GPX, and RuBisCO showed negative values. Photosynthetic pigments (chlorophylls and carotenoids), PEPC, flavonoids, RuBisCO, PAL, and yellow pigments contributed significantly to the plant’s response.

UAF was closely associated with APX and phenols, suggesting changes in metabolic activity and antioxidant defense mechanisms. The SD and SFD treatments were located near Flav and Chl a/b, indicating a relationship with oxidative stress processes. CK was linked to GSH. UAFD was primarily associated with GPX and MDA levels. Similar to UAD, it showed a strong association with H_2_O_2_. Notably, UAF treatment may be associated with the maintenance of metabolic activity and potentially increased carbon fixation, whereas UAFD is associated with increased oxidative stress. It has been reported that UA applied to soil reduces microbial biomass, acid protease and acid phosphatase activity, and the abundance of the eukaryotic 18S ribosomal RNA gene [22], so foliar application would be the most viable alternative. The maintenance of photosynthetic activity following foliar application of UA can be explained by the fact that this metabolite has properties that protect against high light intensity [15], and the applied dose does not inhibit photosystem II (PSII) and acts as a promoter and activator of the antioxidant system [18], which in turn improves biomass accumulation [21].

## 3. Materials and Methods

### 3.1. Biological Materials

The biological material used consisted of “Albión” strawberry seedlings grown in 4 L polyethylene bags containing a substrate mixture of peat moss and perlite in a 1:1 (*v*/*v*) ratio. Fertilization was provided by applying a Steiner solution [46] with an electrical conductivity of 1.5 and a pH of 6.5.

### 3.2. Preparation and Application of Usnic Acid

UA was prepared according to the method described by Lechowski et al. [12]. Briefly, 640 mg of (+) UA (reagent grade, 98% purity, Sigma Aldrich Inc., St. Louis, MO, USA, 329967-5G) was weighed and dissolved in 40% NaOH (Jalmek, San Nicolas de los Garza, Nuevo León, Mexico) solution. The pH was adjusted to 7.18 ± 0.5, and a dispersant nonylphenol (10) polyoxyethylene was added to the mixture. The UA solution was prepared at a concentration of 400 μg/mL. The UA solution was applied to strawberry plants at a dose of 15 mL/plant. The first application was made at stage 18 of the BBCH scale (8–9 expanded leaves) at 15-day intervals. This resulted in a total of three applications: 45 mL/plant.

### 3.3. Treatments

The concentration of usnic acid applied was 400 µg/mL [47]. The treatments evaluated were usnic acid applied foliar (UAF), drench application of usnic acid (UAD), foliar + drench application of usnic acid (UAFD), foliar application of solvent (40% NaOH) (SF), drench application of solvent (SD), drench + foliar application of solvent (SFD), and a control with distilled water (CK).

### 3.4. Sampling and Sample Preparation

Leaf tissue samples were collected 117 days after transplantation at the BBCH stage 55 (first floral primordia in the leaf rosette). Five physiologically mature leaves were collected, stored on ice, and subsequently frozen at −20 °C. Finally, the leaf tissue was freeze-dried (Freeze Dryer, model ECO-FD10PT, Biobase Meihua Trading Co., Ltd., Jinan, China) for 48 h at 00001 pa. Once freeze-dried, the plant tissue was ground in porcelain mortars and stored until further use. The biochemical parameters (photosynthetic pigments, vitamin C, flavonoids, total phenols, reduced glutathione, glutathione peroxidase, ascorbate peroxidase, phenylalanine ammonia-lyase, catalase, RuBisCO, PEP carboxylase, hydrogen peroxide, malondialdehyde, and proline) and total protein content were quantified using spectrophotometry (ME-UV1800 spectrophotometer; MesuLab Instruments Co., Ltd., Guangzhou, China). The absorbance was recorded at specific wavelengths for each compound.

### 3.5. Content of Photosynthetic Pigments

The contents of chlorophyll a, b, and total chlorophyll, as well as the chlorophyll a/b ratio and β-carotene, were quantified according to Nagata & Yamashita [48]. For extraction, 10 mg of freeze-dried leaf tissue was mixed with 2 mL of hexane–acetone (Jalmek, San Nicolas de los Garza, Nuevo León, Mexico) (3:2), vortexed for 5–10 s, sonicated (Sonicator Baku BK 2000, Guangzhou, China) for 5 min, and centrifuged (Microcentrifuge Frontier FC5515R, Ohaus Corporation, Parsippany, NJ, USA) at 15,000× *g* for 10 min at 4 °C. The supernatant was used to measure absorbance at 645 and 663 nm for chlorophylls and 453, 505, 645, and 663 nm for carotenoids. The values obtained were used in Equations (1) and (2) to calculate the chlorophyll content and in Equation (3) for β-carotene.

(1) Chlorophyll a=0.999×A663−0.0989×A645

(2) Chlorophyll b=0.328×A663+1.77×A645

(3) β-carotene=0.216×A663−1.22×A645−0.304×A505+0.452×A453

### 3.6. Yellow Pigments

The yellow pigment (β-cryptoxanthin and zeaxanthin) content was determined according to the methodology described by Hornero-Méndez & Minguez-Mosquera [49]. To do this, a mixture of hexane and acetone (3:2) was added to 10 mg of leaf tissue, vortexed for 5 s, sonicated (5 min), and centrifuged (for 10 min at 15,000× *g* and 4 °C). The absorbance of the supernatant was measured at 472 nm wavelength. To calculate the pigment content, the absorbance values were used in Equation (4).

(4) Yellow carotenoids=(1724.3×A472−2450.1×A508)/270.9

### 3.7. Determination of Vitamin C Content

Vitamin C was quantified using the method described by Hung & Yen [50]. Ten milligrams of leaf tissue were mixed with 1 mL of 1% (*w*/*v*) metaphosphoric acid (Sigma Aldrich Inc., St. Louis, MO, USA). The homogenate was vortexed and filtered. The filtrate (75 µL) was mixed with 675 µL of 2,6-dichlorophenolindophenol (50 µM) (Sigma Aldrich Inc., St. Louis, MO, USA) and allowed to react at room temperature for 15 s. For the blank, the following were used: 75 μL of metaphosphoric acid and 675 μL of 2,6-dichlorophenolindophenol (50 µM), following the same procedure as for the samples. The absorbance was measured at 515 nm.

### 3.8. Flavonoids

The flavonoid content was determined using the method described by Arvouet-Grand et al. [51]. For this purpose, 20 mg of freeze-dried leaf tissue was homogenized in 800 µL of methanol (Jalmek, San Nicolas de los Garza, Nuevo León, Mexico). The mixture was then filtered. To 500 µL of the filtrate, 1 mL of a 2% AlCl_3_ (Jalmek, San Nicolas de los Garza, Nuevo León, Mexico) solution was added, and the mixture was allowed to stand for 20 min in the dark. The absorbance was measured at 415 nm.

### 3.9. Total Phenols

The total phenol content was determined according to Singleton et al. [52]. Freeze-dried leaf tissue (20 mg) was homogenized with 400 µL of water-acetone (1:1) (vortexed for 30 s), sonicated (5 min), and centrifuged (10 min at 12,500 rpm and 4 °C). The supernatant (12.5 µL) was mixed with 50 µL of Folin–Ciocalteu reagent (Sigma Aldrich Inc., St. Louis, MO, USA), 125 µL of sodium carbonate (Jalmek, San Nicolas de los Garza, Nuevo León, Mexico) (20%), and 1.25 mL of cold distilled water. The mixture was vortexed (30 s) and placed in a water bath (45 °C for 30 min). The absorbance was measured at 750 nm.

### 3.10. Enzyme Extract (EE)

EE was prepared the EE, the method described by Leija-Martínez et al. [53]. Briefly, 100 mg of freeze-dried leaf tissue was mixed with 10 mg of polyvinylpyrrolidone (PVP), (Sigma Aldrich Inc., St. Louis, MO, USA) followed by the addition of 1.5 mL of 0.1 M phosphate buffer (pH 7.0–7.2) and homogenized. The mixture was vortexed for 5 s to achieve homogenization. The samples were then sonicated for 5 min and centrifuged at 12,500 rpm for 10 min at 4 °C. Finally, the supernatant was collected and filtered. EE was diluted (1:20) and stored at 4 °C. EE was used to determine the levels of reduced glutathione (GSH), glutathione peroxidase (GPX), ascorbate peroxidase (APX), catalase (CAT), and phenylalanine ammonia-lyase (PAL).

#### 3.10.1. Total Protein Content

The Bradford method was used to determine the total protein content [54]. Briefly, 8 μL of the extract was dispensed into an Eppendorf tube, followed by the addition of 400 μL of Bradford reagent. The mixture was then incubated for 10 min at 25 °C, and the absorbance was measured at 595 nm using a spectrophotometer.

#### 3.10.2. Reduced Glutathione (GSH)

To quantify GSH, 120 µL of EE was mixed with 550 µL of Na_2_HPO_4_ (Jalmek, San Nicolas de los Garza, Nuevo León, Mexico) (0.32 M) and 80 µL of dithio-bis-2-nitrobenzoic acid (DTNB, 1 mM diluted in phosphate buffer) (Sigma Aldrich Inc., St. Louis, MO, USA). The homogenate was vortexed, and the absorbance was measured at 412 nm [55].

#### 3.10.3. Glutathione Peroxidase (GPX)

GPX enzymatic activity was determined using the method described by Xue et al. [55] with H_2_O_2_ (Jalmek, San Nicolas de los Garza, Nuevo León, Mexico) as the substrate. A reaction mixture was prepared containing 100 µL of EE, 200 µL of GSH (Sigma Aldrich Inc., St. Louis, MO, USA) (0.01 mM diluted in phosphate buffer), and 100 µL of Na_2_HPO_4_ (0.067 M). The mixture was then incubated in a water bath (25 °C) for 5 min. Subsequently, 100 µL of H_2_O_2_ (1.3 mM) was added, and the reaction was maintained for 10 min at 26 °C. The reaction was stopped by adding 500 µL of trichloroacetic acid (1%) (Sigma Aldrich Inc., St. Louis, MO, USA), followed by a cold bath for 30 min and centrifugation (10,000 rpm for 10 min at 4 °C). Finally, 120 µL of the supernatant was homogenized with 550 µL of Na_2_HPO_4_ (0.32 M) and 80 µL of DTNB (5,5′-dithio-bis-[2-nitrobenzoic acid]) (1 mM), and the absorbance was measured at 412 nm.

#### 3.10.4. Ascorbate Peroxidase (APX)

APX activity was determined using a two-step procedure [56] as follows: At time 0, a reaction mixture was prepared using 25 µL of EE, 125 µL of ascorbate (10 mg/mL), 100 µL of 5% sulfuric acid (H_2_SO_4_) (Jalmek, San Nicolas de los Garza, Nuevo León, Mexico), and 250 µL of H_2_O_2_. The absorbance was measured immediately at 266 nm. The reaction mixture for time 1 was prepared by mixing 25 µL of EE, 125 µL of ascorbate (Jalmek, San Nicolas de los Garza, Nuevo León, Mexico) (10 mg/L), and 250 µL of H_2_O_2_. The mixture was shaken for 1 min, and 100 µL of H_2_SO_4_ (5%) was added. The absorbance was measured at 266 nm.

#### 3.10.5. Phenylalanine Ammonia-Lyase (PAL)

PAL activity was assessed as described by Sykłowska-Baranek et al. [57]. To do this, a reaction mixture was prepared containing 25 µL of EE and 225 µL of phenylalanine (Jalmek, San Nicolas de los Garza, Nuevo León, Mexico) (6 mM in phosphate buffer). The mixture was incubated in a water bath (40 °C for 30 min). The reaction was stopped by adding 62.5 µL of hydrochloric acid (Jalmek, San Nicolas de los Garza, Nuevo León, Mexico) (5 N HCl) and cooling in an ice bath for 2 min. Finally, 1.25 mL of distilled water was added, and the absorbance was measured at 290 nm.

#### 3.10.6. Catalase (CAT)

CAT activity was measured at two time points, according to the method described by Dhindsa et al. [58]. At time 0, 50 µL of EE, 200 µL of H_2_SO_4_ (5%), and 500 µL of H_2_O_2_ (100 mM) were mixed. The mixture was vortexed and immediately measured at 270 nm. The reaction mixture for time 1 was prepared with 50 µL of EE and 500 µL of H_2_O_2_ (100 mM), vortexed for 60 s, and 200 µL of H_2_SO_4_ (5%) was added. Finally, the absorbance was measured at 270 nm. CAT activity was determined based on H_2_O_2_ consumption using the difference in absorbance between times 0 and 1 (T0–T1).

### 3.11. Ribulose 1,5-Bisphosphate Carboxylase-Oxygenase (RuBisCO)

RuBisCO activity was determined according to Khan et al. [59] and Usuda [60] methods. For enzyme extraction, 10 mg samples of freeze-dried leaf tissue were homogenized by vortexing with 300 µL of cold extraction buffer [0.25 M Tris-HCl (pH 7.8), 0.05 M MgCl_2_, 0.0025 M EDTA, and 37.5 mg/L dithiothreitol (DTT) (Sigma Aldrich Inc., St. Louis, MO, USA)]. The homogenates were centrifuged at 15,000 rpm for 15 min at 4 °C. For the reaction mixture, 15 µL of supernatant was mixed with 485 µL of reaction buffer [100 mM Tris-HCl (pH 8.0), 40 mM NaHCO_3_, 10 mM MgCl_2_, 0.2 mM NADH, 4 mM ATP, 0.2 mM EDTA, 5 mM DTT, 1 U glyceraldehyde-3-phosphate dehydrogenase, 1 U phosphoglycerate kinase, and 0.02 mM ribulose-1,5-bisphosphate (RuBP) (Sigma Aldrich Inc., St. Louis, MO, USA)]. The enzyme activity was measured at 340 nm.

### 3.12. Phosphoenolpyruvate Carboxylase (PEPC)

PEPC activity was assessed by enzymatic extraction using 10 mg of homogenized leaf tissue with 140 µL of cold extraction buffer [HEPES-NaOH acid [4-(2-hydroxyethyl)piperazine-1-ethanesulfonic acid] (pH 7.8) 50 mM, 1 mM EDTA, 10 mM DTT, 1% (*w*/*v*) polyvinylpyrrolidone (PVP), 0.1% (*v*/*v*) Triton X-100, and 0.2% (*v*/*v*) protease inhibitor cocktail (Sigma Aldrich Inc., St. Louis, MO, USA)] and centrifuged at 15,000 rpm for 15 min at 4 °C. The enzymatic reaction was performed by mixing 15 µL of the supernatant with 485 µL of reaction buffer [EPPS-NaOH [4-(2-hydroxyethyl)1-piperazine propanesulfonic acid] 100 mM, pH 8.0, MgCl_2_ 20 mM, EDTA 1 mM, NaHCO_3_, NADH 0.2 mM, D-glucose-6-phosphate 5 mM, malate dehydrogenase 12 U, phosphoenolpyruvate 2 mM (Sigma Aldrich Inc., St. Louis, MO, USA)]. PEPC activity was assessed based on NADH consumption at 340 nm [61].

### 3.13. β-Carbonic Anhydrase, βCA

The extract for measuring βCA (units of enzyme activity per gram of dry weight per minute; UE g DW^−1^ min^−1^) was prepared by mixing 10 mg of leaf tissue with 600 µL of extraction buffer at 4 °C [50 mM HEPES-NaOH (pH 8.3), 10 mM DTT, 0.5 mM EDTA, 10% *v*/*v* glycerol, 0.1% *v*/*v* Triton X-100 (Sigma Aldrich Inc., St. Louis, MO, USA)]. The homogenates were centrifuged at 15,000 rpm for 15 min at 4 °C. For the reaction, 250 µL of the supernatant was used, and 15 mL of Tris buffer (pH 8.3 at 23 °C) was added while maintaining constant agitation. The pH was verified to be >8.5, and 5 mL of cold carbonated water was immediately added. The activity was determined in Wilbur–Anderson units based on the time required for the pH to change from 8.3 to 6.3 [62]. The unit of enzymatic activity (UE) was calculated using Formula (5).

(5) $$UE\;activity=\left\{\left(Time\;average\;blank-Time\;average\;sample\right)\left(dilution\;factor\right)\right\}\;/\{\left(Taverage\;sample\right)(vol\;of\;enzyme\;extract)\}$$

### 3.14. Hydrogen Peroxide, H_2_O_2_

The H_2_O_2_ content was determined using 10 mg samples of leaf tissue homogenized with 1000 μL of cold 0.1% trichloroacetic acid and centrifuged at 12,000× *g* for 15 min at 4 °C. For quantification, 125 μL of the supernatant was mixed with 375 μL of phosphate buffer (10 mM, pH 7.0), and 500 μL of KI (Materiales y Abastos Especializados SA de CV, México City, Mexico) (1 M) solution. The absorbance was measured at 390 nm [63].

### 3.15. Malondialdehyde (MDA) Content

To quantify MDA [63], 50 mg of freeze-dried leaf tissue was homogenized with 1.25 mL of trichloroacetic acid (TCA, 0.1%) (Sigma Aldrich Inc., St. Louis, MO, USA) and centrifuged at 10,000 rpm at 4 °C for 15 min. For the reaction mixture, 250 μL of the supernatant was collected and mixed with 500 μL of TCA (20%)–TBA (thiobarbituric acid, 0.5%) (Cayman Chemical Company, Ann Arbor, MI, USA). The mixture was placed in a water bath for 20 min at 100 °C, followed by a cold bath to stop the reaction, and then centrifuged at 6000 rpm at 4 °C for 10 min. The absorbance was measured at 532 and 600 nm. The MDA concentration was calculated using the following formula:

(6) $$MDA=\left(A532-A600\right)\times Weight\left(g\right)\times Vol\;reaction\frac{mL}{\varepsilon }\times 1000$$

### 3.16. Free Proline Content

For proline quantification, we used the method described by Bates [64]. Twenty milligrams of leaf tissue were mixed with 400 μL of sulfosalicylic acid (Sigma Aldrich Inc., St. Louis, MO, USA) (30 g/L). The homogenates were centrifuged at 15,000 rpm at 25 °C for 5 min. For the reaction, 100 μL of filtered supernatant was collected, and 100 μL of ninhydrin-phosphoric acid solution (Sigma Aldrich Inc., St. Louis, MO, USA) (1.25 g of ninhydrin in 30 mL of glacial acetic acid and 20 mL of phosphoric acid) and 100 μL of glacial acetic acid (Jalmek, San Nicolas de los Garza, Nuevo León, Mexico) were added. The mixture was incubated in a water bath at 100 °C for 1 h. The reaction was stopped by placing the mixture in an ice bath for 2 min. Then, 500 μL of toluene was added, and the mixture was vortexed. The absorbance was measured at 520 nm.

### 3.17. Experimental Design and Statistical Analysis

The experimental design was a completely randomized design with seven replicates, with two plants per experimental unit (14 plants per treatment), for a total of 98 plants. For biochemical determinations, five replicates per treatment were used. The data were subjected to analysis of variance (ANOVA) and Kruskal–Wallis rank sum test (*p* < 0.05) based on normality (Shapiro–Wilk) using InfoStat v.2020I software. For variables with a normal distribution, Fisher’s Least Significant Difference test of means (Fisher’s LSD) was performed, while those with a non-normal distribution were subjected to Dunn’s Pairwise Comparison post hoc test. In addition, a principal component analysis was performed (R software, 4.6.0).

## 4. Conclusions

(+) UA at 400 µg/mL enhanced the activity of the antioxidant system in strawberry plants, as evidenced by increased GPX and CAT activity, as well as higher GSH content; however, this response depended heavily on the route of administration. It also enhanced the activity of RuBisCO, PEPC, and βCA, suggesting positive effects on processes associated with CO_2_ assimilation, with no evidence of oxidative stress. These findings suggest that UA may modulate the antioxidant system in strawberry plants. We recommend that the best way to apply AU is via foliar application to avoid negative effects on the microbiome and enzymology of the substrate; however, it is necessary to test new dosages and the number of applications. However, it is necessary to evaluate and validate its performance in plants under stressful conditions (both biotic and abiotic). It is also suggested that alternatives to NaOH as a solvent be explored to optimize its application; such studies should aim to ensure that the solvents are environmentally safe and leave no residues. Likewise, the use of nanotechnology should be explored for this metabolite in the future.

## Figures and Tables

**Table 1:** Photosynthetic pigment content in usnic acid-treated “Albion” strawberry plants.

Treatment	Chl a *	Chl b *	Chl T *	Chl a/b *	β-Carotene *	Yellow Pigments *
UAF	6.34 ± 0.87	2.96 ± 0.26	9.30 ± 1.08	2.14 ± 0.20 ^c^	12.92 ± 4.47	23.89 ± 7.69
UAD	6.44 ± 1.73	2.74 ± 0.74	9.18 ± 2.47	2.35 ± 0.03 ^ab^	15.44 ± 3.63	26.76 ± 6.84
UAFD	6.99 ± 1.07	3.12 ± 0.51	10.12 ± 1.57	2.24 ± 0.09 ^bc^	15.34 ± 2.01	26.95 ± 4.70
SF	6.26 ± 0.84	2.65 ± 0.40	8.91 ± 1.24	2.37 ± 0.07 ^ab^	15.01 ± 1.46	27.49 ± 2.34
SD	7.27 ± 0.43	3.14 ± 0.23	10.40 ± 0.66	2.32 ± 0.03 ^ab^	17.04 ± 0.87	30.09 ± 1.86
SFD	6.57 ± 0.92	2.82 ± 0.40	9.40 ± 1.32	2.33 ± 0.05 ^ab^	15.88 ± 1.77	27.31 ± 3.95
CK	6.16 ± 1.38	2.59 ± 0.63	8.75 ± 2.00	2.39 ± 0.07 ^a^	15.36 ± 2.40	27.19 ± 5.34
*p*–value	0.6685	0.4213	0.6198	0.0044	0.3957	0.7041

UAF = 400 µg/mL of usnic acid applied foliar, UAD = 400 µg/mL of usnic acid applied drench, UAFD = 400 µg/mL of usnic acid applied foliar + drench, SF = solvent (40% NaOH) applied foliar, SD = solvent (40% NaOH) applied drench, SFD = solvent (40% NaOH) applied drench + foliar, CK = control with distilled water. * (mg·100 g^−1^ DW). Data are means ± standard deviation (SD) of five replicates. Different letters in the same vertical column indicated significant differences according to Fisher’s Least Significant Difference (LSD) test (*p* < 0.05).

**Table 2:** Enzyme activity associated with photosynthesis in usnic acid-treated “Albion” strawberry plants.

Treatment	RuBisCO ^†^	PEP-Carboxylase ^†^	β-Carbonic Anhydrase ^‡^
UAF	2.01 ± 0.43 ^b^	1.02 ± 0.31 ^ab^	364.33 ± 97.28 ^a^
UAD	4.13 ± 1.34 ^a^	1.19 ± 0.40 ^a^	183.07 ± 77.27 ^b^
UAFD	2.29 ± 0.49 ^b^	0.81 ± 0.27 ^bc^	342.15 ± 90.00 ^a^
SF	0.97 ± 0.22 ^c^	0.21 ± 0.07 ^d^	69.96 ± 17.82 ^c^
SD	0.81 ± 0.24 ^c^	0.24 ± 0.12 ^d^	156.95 ± 110.84 ^bc^
SFD	2.14 ± 0.61 ^b^	0.58 ± 0.21 ^c^	73.62 ± 18.92 ^c^
CK	2.61 ± 1.29 ^b^	0.81 ± 0.16 ^bc^	70.76 ± 15.83 ^c^
*p*-value	<0.0001	<0.0001	<0.0001

UAF = 400 µg/mL of usnic acid applied foliar, UAD = 400 µg/mL of usnic acid applied drench, UAFD = 400 µg/mL usnic acid applied foliar + drench, SF = solvent (40% NaOH) applied foliar, SD = solvent (40% NaOH) applied drench, SFD = solvent (40% NaOH) applied drench + foliar, CK = control with distilled water. ^†^ = μM CO_2_ fixed mg^−1^ protein min^−1^. ^‡^ = UE g DW^−1^ min^−1^. Data are means ± standard deviation (SD) of five replicates. Different letters in the same vertical column indicated significant differences according to Fisher’s Least Significant Difference (LSD) test (*p* < 0.05).

## Data Availability

The data is available from the corresponding author on request.
